# Genetic Variants Associated with Episodic Ataxia in Korea

**DOI:** 10.1038/s41598-017-14254-7

**Published:** 2017-10-23

**Authors:** Kwang-Dong Choi, Ji-Soo Kim, Hyo-Jung Kim, Ileok Jung, Seong-Hae Jeong, Seung-Han Lee, Dong Uk Kim, Sang-Ho Kim, Seo Young Choi, Jin-Hong Shin, Dae-Seong Kim, Kyung-Pil Park, Hyang-Sook Kim, Jae-Hwan Choi

**Affiliations:** 1 Department of Neurology, Pusan National University Hospital, Pusan National University School of Medicine and Biomedical Research Institute, Busan, South Korea; 2 Department of Neurology, Seoul National University Bundang Hospital, Seongnam, South Korea; 3 Research Administration Team, Seoul National University Bundang Hospital, Seongnam, South Korea; 4 Department of Neurology, Korea University Ansan Hospital, Ansan, South Korea; 5 Department of Neurology, Chungnam National University Hospital, Daejeon, South Korea; 6 Department of Neurology, Chonnam National University Hospital, Gwangju, South Korea; 7 Department of Neurology, Malgeunmeori Neurology Center, Gwangju, South Korea; 8 Department of Neurology, Dong-A National University Hospital, Busan, South Korea; 9 Department of Neurology, Pusan National University School of Medicine, Research Institute for Convergence of Biomedical Science and Technology, Pusan National University Yangsan Hospital, Yangsan, Korea

## Abstract

Episodic ataxia (EA) is a rare neurological condition characterized by recurrent spells of truncal ataxia and incoordination. Five genes (*KCNA1*, *CACNA1A*, *CACNB4*, *SLC1A3*, and *UBR4*) have been linked to EA. Despite extensive efforts to genetically diagnose EA, many patients remain still undiagnosed. Whole-exome sequencing was carried out in 39 Korean patients with EA to identify pathogenic mutations of the five known EA genes. We also evaluated 40 candidate genes that cause EA as a secondary phenotype or cerebellar ataxia. Eighteen patients (46%) revealed genetic information useful for establishing a molecular diagnosis of EA. In 11 patients, 16 pathogenic mutations were detected in three EA genes. These included nine mutations in *CACNA1A*, three in *SLC1A3*, and four in *UBR4*. Three patients had mutations in two genes, either *CACNA1A* and *SLC1A3* or *CACNA1A* and *UBR4*, suggesting that *SLC1A3* and *UBR4* may act as genetic modifiers with synergic effects on the abnormal presynaptic activity caused by *CACNA1A* mutations. In seven patients with negative results for screening of EA genes, potential pathogenic mutations were identified in the candidate genes *ATP1A2*, *SCN1A*, *TTBK2*, *TGM6*, *FGF14*, and *KCND3*. This study demonstrates the genetic heterogeneity of Korean EA, and indicates that whole-exome sequencing may be useful for molecular genetic diagnosis of EA.

## Introduction

Episodic ataxia (EA) is a clinically heterogeneous group characterized by recurrent spells of truncal ataxia and incoordination^1–3^. Incidence is likely less than 1/100,000. Most cases have autosomal dominant inheritance, although some sporadic cases have been reported. There are eight subtypes according to clinical features and genetic etiologies. The most common subtypes are EA1 and EA2, caused by mutations involving potassium (*KCNA1*) and calcium (*CACNA1A*) channel genes, respectively. They have well-defined clinical features and have been reported in ethnically different families. EA1 is characterized by brief episodes of ataxia and constant myokymia, whereas EA2 usually presents with longer episodes of ataxia with interictal nystagmus. Onset is typically in early childhood, and episodes are commonly triggered by physical and emotional stress. In EA2, acetazolamide may reduce frequency and severity of attacks. Mutations in *CACNB4* and *SLC1A3* cause EA5 and EA6, respectively, but have been reported in only one or two families^4–6^. In the remaining three subtypes, known EA genes were excluded (EA4) or distinct disease loci were mapped (EA3 and EA7)^7–9^. A recent whole-exome sequencing (WES) study revealed the association of *UBR4* with EA8^10^.

To date, more than 100 different mutations have been described in all EA genes, but molecular diagnostic rates range from 13% to 40%^11–13^. A significant proportion of EA patients do not have mutations in the known EA genes. This suggests a genetic heterogeneity of EA and presence of additional causative genes. EA belongs to a group of neurological channelopathies manifesting as various episodic neurological symptoms and signs^1^. So far, the majority of genetically-confirmed EA have been identified in Caucasian families^11,14–16^. Only a few Asians have been reported, probably due to limited availability of commercial genetic tests^1,17,18^. However, recent advances in next-generation sequencing techniques, such as WES, will facilitate identification of new mutations associated with EA.

In this study, we conducted next-generation sequencing in Korean EA patients to identify pathogenic mutations of five known EA genes (*KCNA1*, *CACNA1A*, *CACNB4*, *SLC1A3*, and *UBR4*), and explored candidate genes that cause EA as a secondary phenotype or cerebellar ataxia.

## Results

After variant filtering, annotation, and interpretation, 204 different variants were identified in the protein-coding regions of five EA genes and 40 candidate genes (Supplementary Table S1).

**Figure 1:**
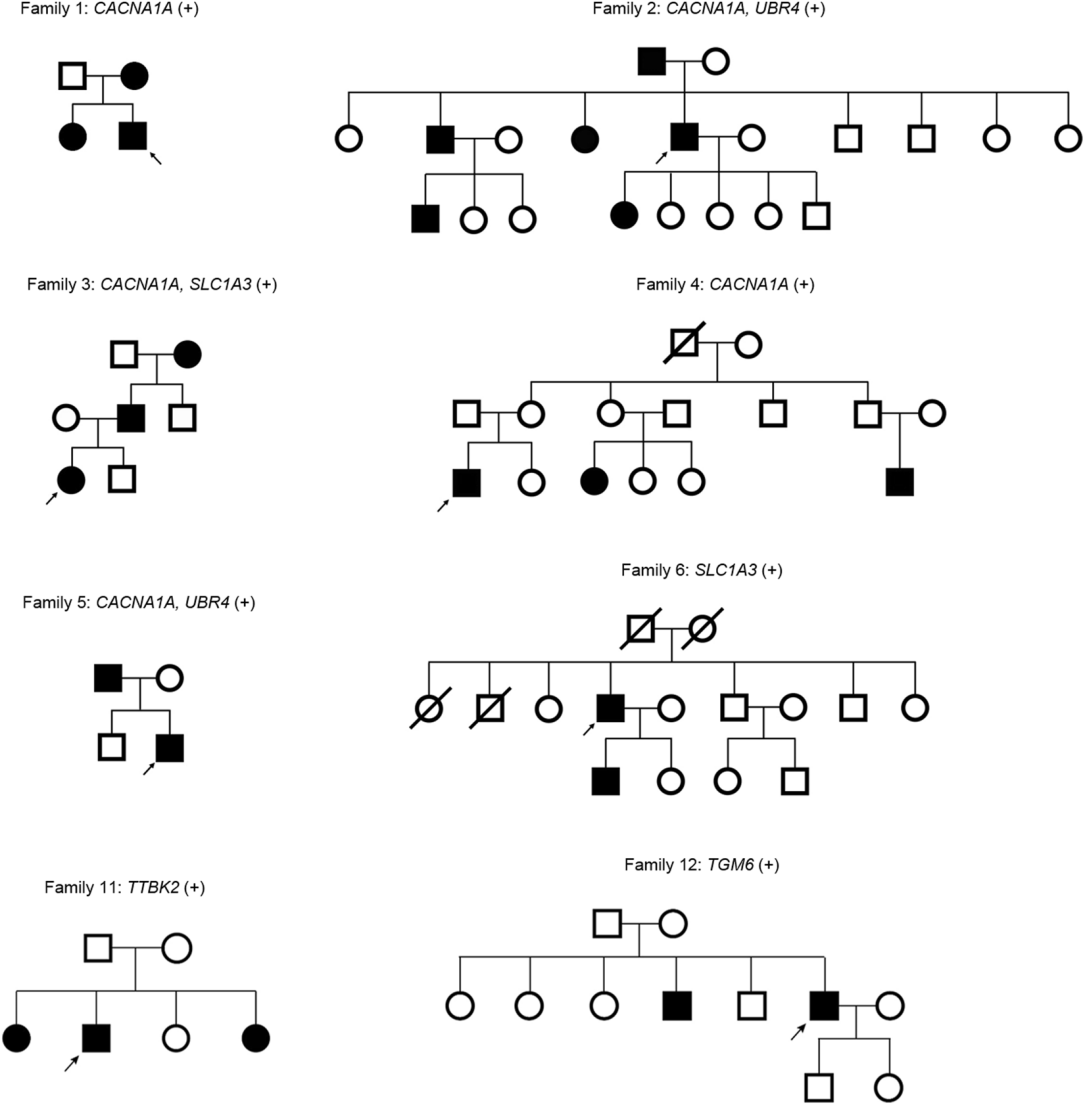
Pedigree of eight families with pathogenic mutations. Solid symbols (squares = males, circles = females) indicate clinically affected individuals; open symbols, unaffected individuals; and slashed symbols, deceased individuals. Probands are indicated by arrows.

We used a stratified approach to identify disease-causing variants. Variants were regarded as *probable* pathogenic mutation when 1) variants were previously reported as a disease-causing mutation, 2) protein truncation was caused via a frameshift or stop codon, or 3) nonsynonymous missense variants were predicted as damaging by at least three of four prediction tools, were present below MAF 0.001, involved a conserved nucleotide position based on a positive GERP score, and segregated with at least one additional affected family member. Nonsynonymous missense variants that met two or more of these criteria, but not all, were considered as *possible* pathogenic mutations. Using this method, we identified 23 pathogenic mutations in 18 of 39 patients (46%, Tables 1 and 2). All variants were absent in 150 unrelated in-house controls. Thirteen of 23 identified mutations were also absent from the inspected exome or genome database. The other 10 were present with a MAF ≤0.0002. Sixteen mutations were found in three EA genes and another seven in three candidate genes. Eight patients had a family history of EA (Fig. 1), while the remaining 10 were sporadic.

**Table 1:** Clinical characteristics of the patients with genetically-confirmed EA.

Patient No	Sex/age	Age of onset	Genetic variants	Duration	Ictal symptoms	Interictal nystagmus	Additional features	Response to acetazolamide
Family Hx. (+)								
1	M/27	19	*CACNA1A*	minutes-hours	ataxia, vertigo, dysarthria, diplopia	DB	(−)	(+)
2	M/48	teenage	*CACNA1A UBR4*	hours	ataxia, dysarthria	DB	cerebellar atrophy	NP
3	F/4	3	*CACNA1A SLC1A3*	hours	ataxia, dizziness	GEN	seizure	(+)
4	M/21	12	*CACNA1A*	hours	ataxia, vertigo, headache	DB	migraine	(+)
5	M/41	9	*CACNA1A UBR4*	hours	ataxia, dizziness, dysarthria	GEN	migraine	(+)
6	M/62	55	*SLC1A3*	hours	ataxia, dizziness, dysarthria	DB	mild truncal ataxia	(+)
11	M/68	53	*TTBK2*	hours	ataxia, vertigo	(−)	(−)	(−)
12	M/56	54	*TGM6*	hours	ataxia, vertigo, dysarthria	GEN	(−)	(−)
Family Hx. (−)								
15	M/39	34	*CACNA1A*	seconds-minutes	dizziness	GEN	(−)	(−)
16	M/33	33	*CACNA1A*	hours	ataxia, vertigo, headache, tinnitus	GEN	(−)	(−)
19	F/49	47	*ATP1A2*	days	ataxia, vertigo, dysarthria, weakness	(−)	intermittent LOC, migraine	(+)
22	F/37	30	*SCN1A*	hours	dizziness, headache	DB	migraine	(−)
23	M/45	38	*SLC1A3*	hours	ataxia, dizziness, dysarthria	GEN	rebound upbeat nystagmus, cognitive impairment	(−)
26	F/54	53	*UBR4*	seconds-minutes	dizziness	GEN	(−)	NP
28	F/52	49	*UBR4*	hours	ataxia, vertigo, tinnitus	(−)	(−)	NP
30	F/62	60	*SCN1A*	hours	vertigo, diplopia	GEN	(−)	(−)
32	M/46	39	*FGF14*	hours	dizziness, headache	DB	(−)	(+)
33	F/17	17	*KCND3*	minutes-hours	ataxia, vertigo, dysarthria	GEN	earfullness	NP

M = male; F = female; GEN = gaze-evoked nystagmus; DB = downbeat nystagmus; LOC = loss of consciousness; NP = not performed.

**Table 2:** Potentially pathogenic variants identified by whole-exome sequencing in 18 EA patients.

Patient No	Family History	Gene	Exon	mRNA	Protein	Variant effect	SIFT	LRT	Polyphen	Mutation taster	GERP	MAF	Pathogenic
1	+	*CACNA1A*	16	c.2030 G > A	p.Gly677Glu	missense	D	D	B	D	4.58	—	possible
2	+	*CACNA1A*	40	c.5956 C > T	p.Gln1986*	nonsense	—	—	—	—	—	—	probable
		*UBR4*	100	c.14630 A > G	p.Tyr4877Cys	missense	D	D	D	D	5.75	—	possible
3	+	*CACNA1A*	23	c.3871_3873delGAG	p.Glu1294 DEL	deletion	—	—	—	—	—	—	probable
		*SLC1A3*	7	c.985 G > A	p.Ala329Thr	missense	D	D	P	D	5.71	0.0003^†^	probable
4	+	*CACNA1A*	5	c.742 T > A	p.Tyr248Asn	missense	D	D	D	D	5.55	—	possible
5	+	*CACNA1A*	32	c.5005 C > T	p.Arg1669*	nonsense	—	—	—	—	—	—	probable
		*CACNA1A*	19	c.2992 G > C	p.Glu998Gln	missense	T	N	B	D	1.91	—	possible
		*UBR4*	83	c.12332 G > A	p.Arg4111His	missense	T	D	P	D	5.25	0.0001^†^	possible
6	+	*SLC1A3*	8	c.1177 G > A	p.Val393Ile	missense	T	D	P	D	5.8	—	probable
11	+	*TTBK2*	15	c.3467 G > A	p.Arg1156Gln	missense	D	D	D	D	5.13	0.00002^†^	possible
12	+	*TGM6*	10	c.1478 C > T	p.Pro493Leu	missense	T	N	D	D	4.67	0.0001^†^	possible
15	−	*CACNA1A*	20	c.3321_3322insC	p.Gly1108Argfs40*	insertion	—	—	—	—	—	—	probable
		*CACNA1A*	46	c.6605_6616delACC AGGAGCGGG	p.Asp2202_Arg2205DEL	deletion	—	—	—	—	—	0.0005^†^	possible
16	−	*CACNA1A*	29	c.4645 C > T	p.Arg1549*	nonsense	—	—	—	—	—	—	probable
19	−	*ATP1A2*	6	c.586 C > T	p.Arg196Cys	missense	D	D	D	D	5.11	0.00003^†^	possible
22	−	*SCN1A*	26	c.5516 T > G	p.Ile1839Ser	missense	D	D	D	D	5.6	0.000008^†^	possible
23	−	*SLC1A3*	7	c.952 A > G	p.Thr318Ala	missense	T	D	B	D	6.07	0.0002^†^	possible
26	−	*UBR4*	103	c.15125 C > T	p.Ala5042Val	missense	T	D	D	D	5.39	0.0002^‡^	possible
28	−	*UBR4*	52	c.7742 C > T	p.Ala2581Val	missense	D	D	D	D	5.95	—	possible
30	−	*SCN1A*	11	c.1688T > A	p.Leu563Gln	missense	D	D	D	D	5.59	—	possible
32	−	*FGF14*	1	c.31 A > G	p.Thr11Ala	missense	D	N	B	D	4.73	—	possible
33	−	*KCND3*	4	c.1291 C > T	p.Arg431Cys	missense	D	D	D	D	5.25	0.000009^†^	possible

Transcript ID: *ATP1A2*, NM_000702.3 (NP_000693.1); *CACNA1A*, NM_023035.2 (NP_075461.2); *FGF14*, NM_175959.2 (NP_787125.1); *KCND3*, NM_004890.4(NP_004971.2); *SCN1A*, NM_006920.4 (NP_008851.3); *SLC1A3*, NM_004172.4 (NP_00416.3); *TGM6*, NM_198994.2 (NP_945345.2); *TTBK2*, NM_173500.3 (NP_775771.3); *UBR4*, NM_020765.2 (NP_065816.2). SIFT- D (damaging), T (tolerated); LRT- D (deleterious), N (neutral); Polyphen- D (probably damaging), P (possibly damaging), B (benign); MutationTaster- D (disease_causing). ^†^MAF based on the Exome Aggregation Consortium (ExAC), ^‡^MAF based on the 1000 Genomes Project.

### Pathogenic mutations identified in previously known EA genes

**Figure 2:**
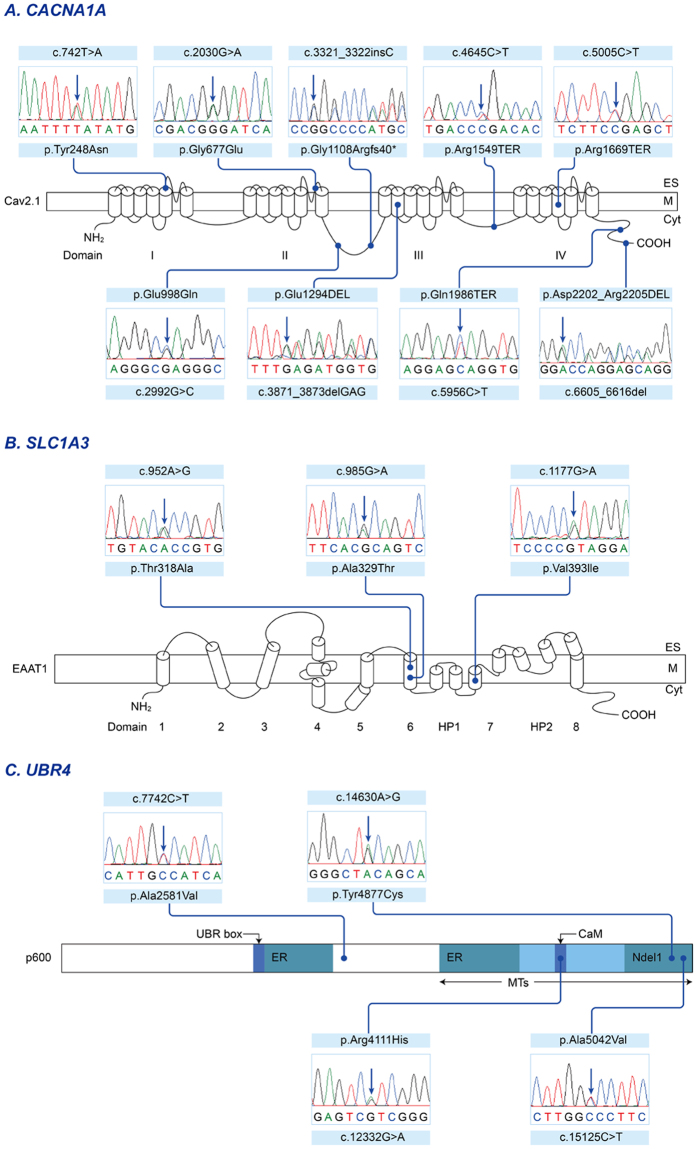
Sequencing results and localization of the mutations in EA genes. (**A**) The Cav2.1 encoded by *CACNA1A* has four homologous domains (I-IV), each consisting of six transmembrane segments (S1-S6) and an additional pore loop located between S5 and S6. Three non-sense mutations (p.Arg1549TER of patient 16, p.Arg1669TER of patient 5 and p.Gln1986TER of patient 2) are predicted premature termination in the S4 of Domain IV or the C-terminal domain. A heterozygous insertion mutation is located in the intracellular linker connecting Domain II and III, and lead to a frameshift and premature termination (p.Gly1108Argfs40TER of patient 15). Two missense mutations (p.Gly677Glu of patient 1 and p.Tyr248Asn of patient 4) involve the pore region (S5-S6) of Domain II and I, respectively. The other missense mutation (p.Glu998Gln of patient 5) is located in the intracellular linker. The in-frame deletions without truncation of the protein (p.Glu1294DEL of patient 3 and p.Asp2202_Arg2205DEL of patient 15) are in the S2 of Domain III and the C-terminal domain, respectively. (**B**) The EAAT1 encoded by *SLC1A3* is composed of eight alpha-helical transmembrane domains (TMDs) and re-entrant hairpin loops (HP) 1 and 2 flanking TMD7. The first sixth TMDs form a scaffold that surrounds a C-terminal core domain comprising HP1, TMD7, HP2, and TMD8. Two missense mutations (p.Ala329Thr of patient 3 and p.Thr318Ala of patient 23) are located in TMD6, while the other mutation (p.Val393Ile of patient 6) is located in TMD7, the critical binding site for glutamate and various coupled ions, Na^+^, H^+^ and K^+^. (**C**) The p600 protein encoded by *UBR4* contains several identified functional domains including UBR box, microtubule (MT)-binding domains, and calmodulin (CaM)-binding domain. The two endoplasmic reticulum (ER)-binding regions are located near the center of the protein and within the MT-binding domain. The Ndel1-binding region overlaps with the MT-binding domain. Three missense mutations (p.Arg4111His of patient 5, p.Tyr4877Cys of patient 2, and p.Ala5042Val of patient 26) are located within the MT-binding domain, and one (p.Arg4111His) involves the CaM-binding domain interacting with calmodulin. The other one (p.Ala2581Val of patient 28) is situated in the functionally-unknown region.

Of the previously known EA genes, seven *probable* and nine *possible* pathogenic mutations were identified in six families and five sporadic cases (Table 2). These included nine mutations in *CACNA1A* (five *probable* and four *possible*; Fig. 2A), three in *SLC1A3* (two *probable* and one *possible*; Fig. 2B), and four in *UBR4* (all *possible*; Fig. 2C). Three patients (patient 2, 3 and 5) had mutations in two genes, either *CACNA1A* and *SLC1A3* or *CACNA1A* and *UBR4*.

In patient 5, two *CACNA1A* and one *UBR4* mutations were detected. One was a nonsense mutation in *CACNA1A* (exon 32, c.5005 C > T, p.Arg1669TER), predicted to encode a truncated protein within Domain IV of the Cav2.1 α1 subunit. The remaining two missense mutations (*CACNA1A* exon 19, c.2992 G > C, p.Glu998Gln; *UBR4* exon 83, c.12332 G > A, p.Arg4111His) were either absent in dbSNP147 or had a MAF of 0.0001 in the ExAC Browser, and so were designated as *possible* pathogenic mutations. Patient 2 also had a nonsense mutation in *CACNA1A* (exon 40, c.5956 C > T, p.Gln1986TER) that predicted a premature termination within the C-terminal domain. The patient had an additional novel missense mutation in *UBR4* (exon 100, c.14630 A > G, p.Tyr4877Cys). Patient 3 had *probable* pathogenic mutations in *CACNA1A* and *SLC1A3*. One was an in-frame deletion of one amino acid due to a three-nucleotide deletion in *CACNA1A* (exon 23, c.3871_3873del GAG, p.Glu1294DEL) and the other was a *SLC1A3* missense mutation in highly conserved amino acid (exon 7, c.985 G > A, p.Ala329Thr). Both mutations were detected in the affected father, but not in the unaffected mother. Patient 1 and 4 had a novel missense mutation in *CACNA1A* (exon 16, c.2030 G > A, p.Gly677Glu; exon 5, c.742 T > A, p.Tyr248Asn). Mutations involved the pore loop region of Domain II and Domain I, respectively. They changed the highly conserved amino acid and were predicted to be pathogenic with at least three of the prediction tools. However, both were regarded as *possible* pathogenic mutations because allelic segregation could not be confirmed due to limited availability of additional family members. Patient 15 had two *CACNA1A* mutations. One was a heterozygous insertion mutation that led to frameshift and premature termination (exon 20, c.3321_3322insC, p.Gly1108Argfs40TER). The other was an in-frame deletion of four amino acid (exon 46, c.6605_6616delACCAGGAGCGGG, p.Asp2202_Arg2205DEL) that has been reported as a rare variant^19^. Patient 16 had the previously reported disease-causing mutation in *CACNA1A* (exon 29, c.4645 C > T, p.Arg1549TER)^20^.

Two *SLC1A3* mutations were detected in one family (patient 6) and sporadic case (patient 23). A missense mutation (exon 8, c.1177 G > A, p.Val393Ile) of patient 6 was designated as a *probable* pathogenic mutation based on segregation within an affected family member, absence in public databases, positive GERP score, and deleterious effect by three prediction tools. Patient 23 had another missense mutation (exon 7, c.952 A > G, p.Thr318Ala) in a highly conserved residue with a MAF of 0.0002 in the ExAC Browser, and was regarded as a *possible* pathogenic mutation.

Two *UBR4* missense mutations were found in two sporadic cases: exon 103, c.15125 C > T, p.Ala5042Val in patient 26; exon 52, c.7742 C > T, p.Ala2581Val in patient 28. Both were either absent in dbSNP147 or had a MAF of 0.0002 in the 1,000 Genomes. They changed a highly conserved amino acid and were predicted to be pathogenic with at least three of the prediction tools, but were regarded as *possible* pathogenic mutations because we could not confirm they arose *de novo*.

### Pathogenic mutations identified in candidate genes

**Figure 3:**
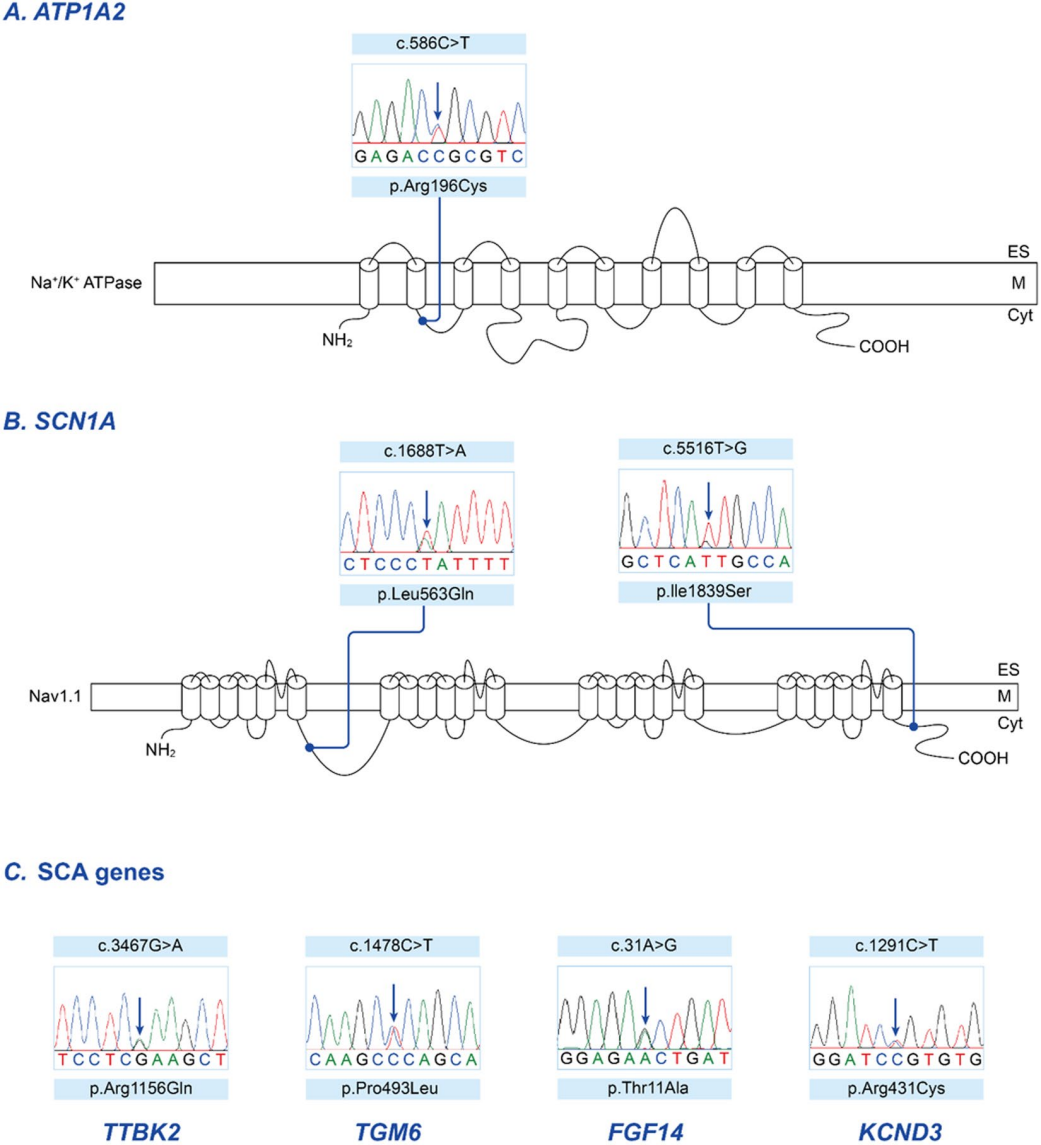
Sequencing results and localization of the mutations in candidate genes. (**A**) The α2 subunit of the Na^+^/K^+^-ATPase encoded by *ATP1A2*, consists of 10 transmembrane segments (M1-M10) with a large cytoplasmic loop between M4 and M5. The missense mutation (p.Arg196Cys of patient 19) is located in the intracellular loop between M2 and M3. This region is associated with A- (actuator) domain responsible for the binding and hydrolysis of ATP. (**B**) The α1 subunit of the Na^+^ channel encoded by *SCN1A*, has four homologous domains (I-IV), each consisting of six transmembrane segments (S1-S6) and an additional pore loop located between S5 and S6. Two missense mutations (p.Ile1839Ser of patient 22 and p.Leu563Gln of patient 30) are located in the C-terminal domain and the intracellular linker connecting Domain I and II, respectively. (**C**) Missense mutations of genes associated spinocerebellar ataxia in four patients with episodic ataxia: *TTBK2 (*exon 15, c.3467 G > A, p.Arg1156Gln in patient 11), *TGM6 (*exon 10, c.1478 C > T, p.Pro493Leu in patient 12), *FGF14 (*exon 1, c.31 A > G, p.Thr11Ala in patient 32), *KCND3 (*exon 4, c.1291 C > T, p.Arg431Cys in patient 33).

Of 28 patients for whom pathogenic mutations could not be identified in the known EA genes, seven had *possible* pathogenic mutations in candidate genes (Table 2). Two had a family history, and the remaining five were sporadic cases. Three patients had a missense mutations in *ATP1A2* (exon 6, c586C > T, p.Arg196Cys in patient 19; Fig. 3A) or *SCN1A* (exon 26, c.5516 T > G, p.Ile1839Ser in patient 22; exon 11, c.1688T > A, p.Leu563Gln in patient 30; Fig. 3B), which are genes associated with FHM. All were predicted to be pathogenic by four prediction tools, and were either absent or displayed a MAF < 0.0001 in the ExAC Browser. Four patients had missense mutation in genes associated with spinocerebellar ataxia, such as *TTBK2 (*exon 15, c.3467 G > A, p.Arg1156Gln in patient 11), *TGM6 (*exon 10, c.1478 C > T, p.Pro493Leu in patient 12), *FGF14 (*exon 1, c.31 A > G, p.Thr11Ala in patient 32), *KCND3 (*exon 4, c.1291 C > T, p.Arg431Cys in patient 33; Fig. 3C). These mutations were either absent in public database or had a MAF < 0.0001.

Additionally, we analyzed rare variants of all genes in the remaining 21 patients to search for putative genes. There were some genes that shared rare variants between individuals; these included *MUC6*, *KCNJ12*, *SIRPA*, *CELA1*, and *DNAH17* (Supplementary Data). However, they could not be considered as new candidate genes associated with EA due to the lack of potential contribution to cerebellar ataxia and the unknown functions of genes.

### Clinical characteristics of patients with pathogenic mutations

Clinical characteristics of the 18 patients with pathogenic mutations were presented in Table 1. They included nine men and seven women with mean age of 42.3 ± 16 years.

Five patients with *CACNA1A* mutation (patients 1, 2, 3, 4, and 5) revealed typical clinical features of EA2 including early age of onset, a positive family history, recurrent ataxia for several hours, interictal nystagmus, and response to acetazolamide. However, the remaining two patients with *CACNA1A* mutation (patients 15 and 16) were sporadic cases and revealed unusual findings including late-onset of age, brief episodes of dizziness, and poor response to acetazolamide. Two patients (patients 4 and 5) had migraine, and one (patient 3) had a history of febrile convulsion. Although three patients had an additional mutation in *SLC1A3* (patient 3) or *UBR4* (patients 2 and 5), there were no distinctive symptoms during attacks.

Two patients with *SLC1A3* mutations (patients 6 and 23) also exhibited typical EA2-like symptoms including recurrent ataxia, slurred speech lasting several hours and interictal nystagmus, but had late-onset in the fourth or sixth decades. Patient 6 showed a mildly progressive baseline ataxia and good response to acetazolamide or 3,4- diaminopyridine. Patient 23 showed mild cognitive impairments and rebound upbeat nystagmus, which developed on resuming the neutral position after leftward or downward gaze. Attacks did not respond to acetazolamide. Two patients with *UBR4* mutation (patients 26 and 28) presented with recurrent vertigo/dizziness and imbalance lasting several hours with interictal nystagmus beginning in their fifth or sixth decades.

**Figure 4:**
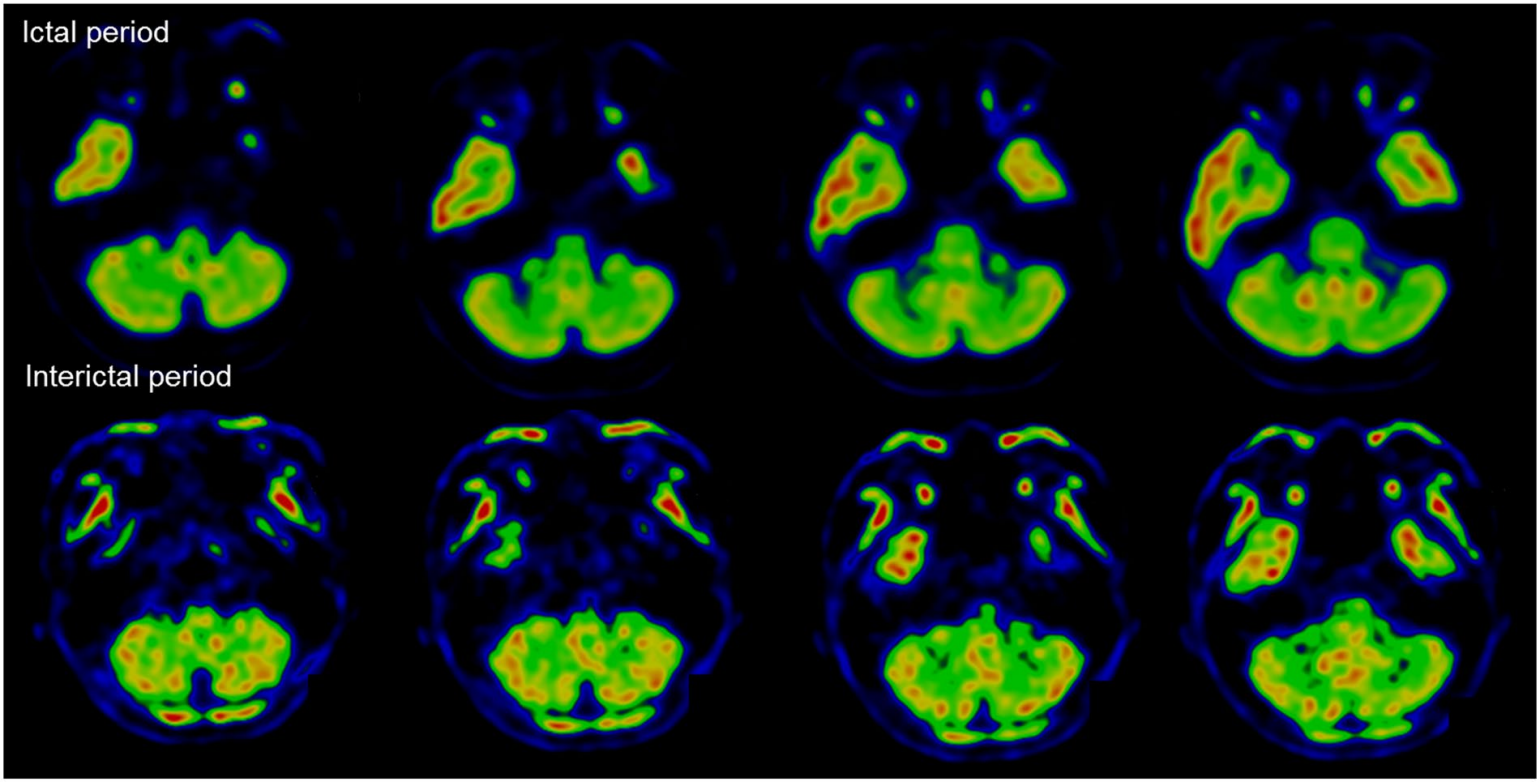
Ictal and interictal cerebellar metabolism in patient 19 with *ATP1A2* mutation. Compared with interictal PET (low panels), ictal images (upper panels) show significant hypometabolism in bilateral cerebellar hemispheres.

Seven patients with mutations in candidate genes presented with recurrent ataxia, vertigo/dizziness, and interictal nystagmus with variable ages at onset. Two of three patients with FHM gene mutations had a history of migraine. Among them, patient 19 with *ATP1A2* mutations had distinctive clinical features. The patient was a 49-year-old woman with recurrent ataxia, dysarthria, vertigo, and limbs weakness provoked by emotional and physical stress since she was 47. She had a history of migraine without visual aura. Between attacks, neurological examinations were unremarkable. During attacks, she showed severe truncal ataxia, dysarthria, quadriparesis of MRC grade 4, and horizontal gaze-evoked nystagmus. Occasionally, she developed decreased consciousness without evidence of seizure on an electroencephalogram. Attacks usually lasted for several days and were resolved in two weeks. Brain magnetic resonance imaging scans were unremarkable, but 18-fluorodeoxyglucose positron emission tomography (FDG-PET) disclosed hypometabolism in both cerebellar hemispheres during attacks, which normalized after one-month (Fig. 4). The attacks were prevented by acetazolamide.

## Discussion

This is the largest genetic analysis study for Korean patients with EA. Using WES, we identified 11 patients with pathogenic mutations in known EA genes and an additional seven with variants of potential pathogenicity in candidate genes. Overall, 46% of patients revealed genetic information useful for establishing molecular diagnosis. These results indicate that WES may be useful for molecular genetic diagnosis of EA, and should be considered in the routine genetic evaluation of patients with EA.

To date, five genes have been linked to EA^1–3^. All except *UBR4* encode ion channel proteins located on the neuronal or glial membrane, and play critical roles in excitatory neurotransmission. In this study, 28% of patients had pathogenic mutations in three EA genes (*CACNA1A, SLC1A3, UBR4*). This is consistent with a molecular diagnosis rate of WES in a large cohort of patients referred for evaluation of suspected genetic diseases^21,22^. Rates varied with clinical phenotype, and the highest was for the specific neurological group^22^. Nevertheless, approximately 70% of patients do not have mutation in the known EA genes, suggesting a genetic heterogeneity of EA and presence of additional causative EA genes.

EA2 is the most common subtype of EA. It is caused by mutations in *CACNA1A* that encodes Cav2.1, the α1 subunit of the P/Q-type voltage-gated Ca^2+^ channel. The α1 subunit is composed of four homologous domains (I-IV), each of which contains six transmembrane segments (S1-S6) and an additional pore loop located between S5 and S6^23^. This channel is widely expressed in Purkinje and granule cells of the cerebellum, and mediates Ca^2+^ entry into cells and regulates the precision of pace-making. Thus, *CACNA1A* mutations lead to decreased Ca^2+^ entry through Cav2.1 and the irregular firing of Purkinje cells. Most mutations in EA2 predict premature truncation of the protein, but some non-truncating mutations have also been reported^12,14,24,25^. Presently, we identified nine mutations in *CACNA1A*. Two of them were previously reported as a disease-causing mutation (p.Arg1549TER) and rare variant (p.Asp2202_Arg2205DEL) respectively^19,20^. The remaining seven were novel mutations, and a missense mutation identified in patient 4 (p.Tyr248Asn) was remarkable because a different missense mutation at the same position had been previously reported (p.Tyr248Cys)^26^. Two patients (patient 5 and 15) had truncating and non-truncating mutation, but each truncating mutation may be strongly associated with loss of channel function. Indeed, the in-frame deletion of patient 15 has been suggested as a potential genetic modifiers in subjects with Dravet syndrome^19^.

Our *CACNA1A* mutations mostly involve critical regions for channel activity. Typically, most *CACNA1A* mutations are widely scattered along the entire gene, but missense mutations usually involve S5-S6 linkers and their borders of each domain. S5-S6 linkers with S5 and S6 form the pore region of the channels, and play a critical role in ion selectivity and permeation of the Ca^2+^ channel^23^. Our two missense mutations (p.Gly677Glu, p.Tyr248Asn) also involved the pore region (S5-S6) of Domain II and I, respectively, that may interrupt channel gating activity. Another two mutations (p.Glu998Gln, p.Gly1108Argfs40TER) were located in the intracellular linker connecting Domain II and III. This loop interacts with SNARE proteins that are essential for docking of synaptic vesicles with the presynaptic membrane^23^. Three nonsense mutations (p.Arg1549TER, p.Arg1669TER, p.Gln1986TER) were localized behind of Domain III and predicted a premature termination within Domain IV or C-terminal domain. The C-terminal domain is a critical region for the Ca^2+^ channel function by G protein regulation and binding of Ca^2+^/Calmodulin, so a truncation of C-terminal may lead to loss of Ca^2+^ channel function. Most truncating mutations including those of this study are preferentially placed in the second half of the α1 subunit, and this suggests the genetic defect may cause loss of function by dominant negative effect^2,11,18,27^. However, the haploinsufficiency hypothesis was also proposed because large deletion or shortest truncating mutation with only Domain I intact cannot have a dominant negative effect on normal allele^11,28^.

EA6 is caused by mutations in *SLC1A3*, that encode excitatory amino acid transporter 1 (EAAT1), a glial glutamate transporter. Since EAAT1 is responsible for glutamate uptake in synapses, the *SLC1A3* mutation leads to excessive extracellular accumulation of glutamate and neurotoxic insults^29^. *UBR4* was recently identified as a candidate gene of EA8^10^. It encodes an ubiquitin-protein ligase that interacts with Ca^2+^ bound calmodulin in the cytoplasm^30^. It may also regulate the release of internal Ca^2+^ stores via an inositol triphosphate receptor associated with SCA 15 and 29^30^. Thus, the *UBR4* mutation may result in abnormal Ca^2+^ signaling within the neuron and development of ataxia. Nevertheless, both EA6 and EA8 have been reported in only a few patients because they have not been widely explored as a usual target for EA screening^5,6,10^. This study identified three *SLC1A3* and four *UBR4* mutations, and most of them involve functional domains of each protein. Interestingly, three were detected along with *CACNA1A* mutation in identical patients. It should be considered that one mutation of the two genes may be a non-pathogenic variant. However, it is also possible that *SLC1A3* and *UBR4* may contribute primarily to the development of EA and act as genetic modifiers with synergic effects on the abnormal presynaptic activity caused by *CACNA1A* mutations. This effect may influence the phenotypic differences or variable penetrances^31^. Roles of modifier genes have been described in several disorders, such as spinal muscular atrophy, retinitis pigmentosa, and familial amyotrophic lateral sclerosis^31^. In this study, patients with only *SLC1A3* or *UBR4* mutations showed typical EA2-like symptoms, but had late-onset of age compared to those with the additional *CACNA1A* mutation. The roles of *SLC1A3* and *UBR4* as modifier genes in EA may require further investigation.

Of the 28 patients without mutations in five EA genes, we additionally detected three variants with potential pathogenicity in FHM genes, such as *ATP1A2* and *SCN1A*. FHM is a rare subtype of migraine with aura. Attacks are associated with transient hemiparesis due to mutations in three genes: *CACNA1A* (FHM1), *ATP1A2* (FHM2), and *SCN1A* (FHM3)^32^. *ATP1A2* encodes the α2 subunit of the Na^+^/K^+^-ATPase^33^, while *SCN1A* encodes the α1 subunit of the Na^+^ channel^34^. Along with other EA genes, they are involved in modulation of ion fluxes in glutamatergic presynaptic terminals, and regulate the release of glutamate in the synapse. The result is a significant clinical overlap between EA and FHM, and many patients with FHM show cerebellar symptoms and signs. EA2 and FHM1 are allelic disorders caused by mutations in *CACNA1A* ^35^. Furthermore, clinical spectrums of *ATP1A2* or *SCN1A* mutations have been expanded to alternating hemiplegia of childhood, basilar migraine, childhood epilepsy, and progressive hearing loss with migraine^36–40^. Recently, a new missense mutation in *ATP1A2* was identified in a patient with highly similar symptoms to EA2^15^. Our patients who had *ATP1A2* or *SCN1A* mutations also showed recurrent ataxia, vertigo/dizziness, and interictal nystagmus without hemiplegic migraine. Especially, one patient with *ATP1A2* mutation had mainly cerebellar symptoms and signs with bilateral cerebellar hypometabolism on FDG-PET during attacks. These findings lead to potential clinical confusion in differential diagnosis of EA from FHM based on genetic study. Thus, screening of FHM genes may be a clinically valuable approach to establish a genetic confirmation of EA.

In addition, we identified rare variants in the genes associated with SCAs; *TTBK2* (SCA 11), *KCND3* (SCA 19), *FGF14* (SCA 27), and *TGM6* (SCA 35). Although SCAs are characterized by progressive cerebellar ataxia, some subtypes may present with fluctuating ataxia like EA. Some patients with SCA6 can show the classic features of EA2 because SCA6 and EA2 are allelic disorders caused by mutation in *CACNA1A* ^1,2^. Furthermore, different phenotypes ranging from SCA6 to EA2 can co-occur within the same family^41,42^. Mutations in *FGF14* have been also reported in autosomal dominant EA and fever-triggered EA^43,44^. Besides SCA genes, many genes encoding the ion channels, transporters, or synaptic proteins play crucial roles in regulating neural excitability in the central nervous system. They are often associated with episodic neurological disorders, such as epilepsy, migraine, and paroxysmal movement disorders. In these disorders, several overlapping syndromes have been described, of which EA has been reported as one of various phenotypes^1^. Recently, late-onset EA has been proposed as the spectrum of *SCN2A*-associated phenotypes^45,46^. In addition, other genes including *ATP1A3, NALCN*, *DARS2*, *SLC2A1*, and *PRRT2* have been also reported as causative genes associated with EA^47–51^. Therefore, extensive searches for these genes may help define novel candidate genes and identify new mutations associated with EA.

This study had several limitations. A selection bias should be considered in interpreting results. Since many patients showed the recurrent episodes lasting several hours, we could not detect *KCNA1* mutation resulting in EA1 characterized by brief episodes of ataxia. We also did not perform a functional study determining pathogenicity of our mutations. Despite the rarity and putative pathogenicity of variants, establishing pathogenicity may be difficult without functional study, especially in sporadic cases. For this reason, *possible* pathogenic mutations were more common in our study. We were unable to identify new casual genes because trio-based exome sequencing could not be performed due to a refusal of inspection in additional family members.

In conclusion, we have identified genetic variants associated with EA in approximately 46% of patients. Our results reveal the significant diagnostic yield of WES to detect mutations in genes causing EA. Since there are still many EA patients without genetic confirmations, further assessments for candidate genes are needed.

## Methods

### Subjects

We recruited 39 unrelated patients with clinically-diagnosed EA from 2012 to 2016 at seven hospitals in Korea. Inclusion criteria were the repetitive spells of ataxia or vertigo/dizziness of variable duration. All patients had no expansions of coding CAG repeats in genes that underline spinocerebellar ataxia 1, 2, 3, 6, 7, 8, and 17. Patients included 19 men and 20 women with age ranging from 4 to 69 years (mean age 41.7 ± 16.0 years). Age of onset varied and ranged from early childhood to the seventh decades (mean age of onset 32.9 ± 17.1 years). Fourteen had a significant family history of EA. All showed recurrent episodes of ataxia or vertigo/dizziness with a clear onset and resolution of symptoms. Episodes usually lasted for several minutes to hours, and were commonly triggered by physical and emotional stress. Twenty-nine had interictal nystagmus, such as either gaze-evoked nystagmus and/or downbeat nystagmus. Of 29 patients treated with acetazolamide, 20 showed decrease in frequency and severity of episodes.

All experiments followed the tenets of the Declaration of Helsinki, and informed consent was obtained after the nature and possible consequences of this study had been explained to the participants. This study was approved by the institutional review boards of Pusan National University Yangsan Hospital. Because the present study included all consecutive patients during the research period, one family previously reported was included in this report^52^.

### Molecular analysis

Genomic DNA was extracted from the blood sample of each patient. Quality and quantity of extracted DNA were measured using NanoDrop® (Invitrogen Life Technologies; Milan, Italy) and Qubit® (Invitrogen Life Technologies) platform. WES was conducted using the Ion Torrent platform (Life Technologies), according to the manufacturer’s specifications. Briefly, 20ng of purified genomic DNA were used for library construction with the Ion AmpliSeq Exome Panel (Life Technologies). Emulsion PCR was conducted with the OneTouch DL system (Life Technologies). Quality of the obtained library was evaluated by qPCR Quantitation (Corbett: QIAGEN). Sequencing was run on the Ion Torrent Proton (Life Technologies) loaded with a P1 chip as per the manufacturer’s protocol. Data analysis including alignment to the hg19 human reference genome and variant calling was conducted using the Torrent Suite Software v.4.2 (Life Technologies). Filtered variants were annotated using both the Ion Reporter software v4.2 (Life Technologies). Variants causing non-synonymous amino acid changes, stop codons, in-frame insertions/deletions in coding regions, or changes to splice site sequences in exon/intron boundaries were identified. Common variants with minor allele frequency (MAF) > 0.001 that represented in dbSNP147, the Exome Aggregation Consortium (ExAC), the 1000 Genomes Project, and the NHLBI GO Exome Sequencing Project (ESP) 6500 were excluded.

### Mutation screening and identification

We adopted a stepwise approach to identify genetic mutation responsible for EA (Supplementary Table). First, we screened for presence of pathogenic variants in EA genes registered on Online Mendelian Inheritance in Man (OMIM). These included *KCNA1* (EA1, OMIM 176260), *CACNA1A* (EA2, OMIM 601011), *CACNB4* (EA5, OMIM 601949), *SLC1A3* (EA6, OMIM 600111), and *UBR4* (EA8, OMIM 609890). If no pathogenic variants were identified in EA genes, we next explored genes known or suggested to cause EA as a part of phenotype in the literature. These included *SCN2A* ^45,46^, *ATP1A3* ^47^, *NALCN* ^48^, D*ARS2* ^49^, *SLC2A1* ^50^, *FGF14* ^43,44^, and *PRRT2* ^51^. Typically, mutations in these genes lead to epilepsy, neurodegenerative disorders, or paroxysmal dyskinesia as the primary phenotype. We also considered genes associated with familial hemiplegic migraine (FHM) as candidates, because *ATP1A2* (FHM2, OMIM 182340) and *SCN1A* (FHM3, OMIM 182389) play a significant role in excitatory neurotransmission with EA genes within the neuronal and glial membranes, and there are significant clinical overlaps between EA and FHM^1,2^. Finally, we screened all known genes associated with cerebellar ataxia based on previous literature and OMIM records^53^. All variants detected by the above process were annotated for previously reported disease-causing variants using the Human Gene Mutation Database (HGMD) and Korean Personal Genome Project (KPGP) information. Pathogenicity of non-synonymous variants was analyzed using the following predictive software: Sorting Intolerant From Tolerant (SIFT), Likelihood Ratio Test (LRT), Polyphen2, and MutationTaster. Conservation at the base position was evaluated using Genomic Evolutionary Rate Profiling (GERP). All variants were further confirmed by PCR-based direct sequencing, and were screened in 150 Korean controls.

## Electronic supplementary material

Supplementary Table 1.

Supplementary Dataset1
