# Supplementary material for: Genetic Variants Associated with Episodic Ataxia in Korea
Source: Sci Rep. 2017 Oct 23;7:13855. doi: 10.1038/s41598-017-14254-7 (PMC5653837; doi:10.1038/s41598-017-14254-7)
Supplement: Supplementary file 1 — Supplementary Table 1. [file 41598_2017_14254_MOESM1_ESM.doc]

**Genetic Variants Associated with Episodic Ataxia in Korea**

Kwang-Dong Choi, MD; Ji-Soo Kim, MD; Hyo-Jung Kim, PhD; Ileok Jung, MD; Seong-Hae Jeong, MD; Seung-Han Lee, MD; Dong Uk Kim, MD; Sang-Ho Kim, MD; Seo Young Choi, MD; Jin-Hong Shin, MD; Dae-Seong Kim, MD; Kyung-Pil Park, MD; Hyang-Sook Kim, MSc;

Jae-Hwan Choi, MD

Supplementary Table 1. Genes screened for episodic ataxia

| Gene | OMIM number | Phenotype | Number of SNVs in coding regions |
| --- | --- | --- | --- |
| **Episodic ataxia genes** | | |  |
| *KCNA1* | 176260 | Episodic ataxia 1 | 5 |
| *CACNA1A* | 601011 | Episodic ataxia 2  Spinocerebellar ataxia 6  Familial hemiplegic migraine-1 (FHM1) | 31 |
| *CACNB4* | 601949 | Episodic ataxia 5 | 1 |
| *SLC1A3* | 600111 | Episodic ataxia 6 | 8 |
| *UBR4* | 609890 | Episodic ataxia 8 | 23 |
| **Candidate genes** | | |  |
| *SCN2A* | 182390 | Early infantile epileptic encephalopathy-11 | 7 |
| *ATP1A3* | 182350 | Alternating hemiplegia of childhood-2 (AHC2)  Cerebellar ataxia, areflexia, pes cavus, optic atrophy, and sensorineural hearing loss (CAPOS) | 2 |
| *NALCN* | 611549 | Congenital contractures of the limbs and face, hypotonia, and developmental dealy (CLIFADHH) | 15 |
| *DARS2* | 610956 | Leukoencephalopathy with brainstem and spinal cord involvement and lactate elevation (LBSL) | 4 |
| *SLC2A1* | 138140 | Dystonia-9  GLUT1 deficiency syndrome | 10 |
| *FGF14* | 601515 | Spinocerebellar ataxia 27 | 1 |
| *PRRT2* | 614386 | Familial infantile convulsions with paroxysmal choreoathetosis (ICCA)  Episodic kinesigenic dyskinesia-1 (EKD1) | 4 |
| *ATP1A2* | 182340 | Alternating hemiplegia of childhood-1 (AHC1)  Familial hemiplegic migraine-2 (FHM2) | 6 |
| *SCN1A* | 182389 | Generalized epilepsy with febrile seizures plus, type 2 (GEFSP2)  Familial febrile seizures-3A (FEB3A)  Familial hemiplegic migraine-3 (FHM3) | 14 |
| *ATXN1* | 601556 | Spinocerebellar ataxia 1 | 7 |
| *ATXN2* | 601517 | Spinocerebellar ataxia 2 | 6 |
| *ATXN3* | 607047 | Spinocerebellar ataxia 3 | 7 |
| *SPTBN2* | 604985 | Spinocerebellar ataxia 5  Autosomal recessive spinocerebellar ataxia 14 | 3 |
| *ATXN7* | 607640 | Spinocerebellar ataxia 7 | 5 |
| *ATXN8* | 613289 | Spinocerebellar ataxia 8 | 0 |
| *ATXN10* | 611150 | Spinocerebellar ataxia 10 | 0 |
| *TTBK2* | 611695 | Spinocerebellar ataxia 11 | 4 |
| *PPP2R2B* | 604325 | Spinocerebellar ataxia 12 | 1 |
| *KCNC3* | 176264 | Spinocerebellar ataxia 13 | 2 |
| *PRKCG* | 176980 | Spinocerebellar ataxia 14 | 0 |
| *ITPR1* | 147265 | Spinocerebellar ataxia 15  Congenital nonprogressive cerebellar ataxia (CNPCA)  Gillespie syndrome (GLSP) | 1 |
| *TBP* | 600075 | Spinocerebellar ataxia 17 | 2 |
| *KCND3* | 605411 | Spinocerebellar ataxia 19  Brugada syndrome 9 | 1 |
| *PDYN* | 131340 | Spinocerebellar ataxia 23 | 1 |
| *AFG3L2* | 604571 | Spinocerebellar ataxia 28  Autosomal recessive spastic ataxia-5 (SPAX5) | 0 |
| *BEAN* | 612051 | Spinocerebellar ataxia 31 | 3 |
| *TGM6* | 613900 | Spinocerebellar ataxia 35 | 4 |
| *NOP56* | 614154 | Spinocerebellar ataxia 36 | 4 |
| *ELOVL5* | 611805 | Spinocerebellar ataxia 38 | 0 |
| *CCDC88C* | 611204 | Spinocerebellar ataxia 40  Autosomal recessive nonsyndromic hydrocephalus-1 (HYC1) | 3 |
| *FXN* | 229300 | Friedreich ataxia | 0 |
| *TTPA* | 600415 | Ataxia with vitamin E deficiency (AVED) | 1 |
| *MTP* | 157147 | Abetalipoproteinemia | 0 |
| *POLG* | 174763 | Alpers syndrome  Neurogastrointestinal encephalopathy (MNGIE),  Sensory ataxic neuropathy, dysarthria, and ophthalmoparesis (SANDO)  Autosomal dominant progressive external ophthalmoplegia (adPEO) | 0 |
| *ATM* | 607585 | Ataxia-telangiectasia | 6 |
| *APTX* | 606350 | Early-onset ataxia with oculomotor apraxia and hypoalbuminemia | 2 |
| *SETX* | 608465 | Autosomal recessive spinocerebellar ataxia-1 (SCAR1)  Juvenile amyotrophic lateral sclerosis | 7 |
| *SACS* | 604490 | Spastic ataxia of the Charlevoix-Saguenay type | 3 |
| *CYP27A1* | 606530 | Cerebrotendinous xanthomatosis | 0 |
| *PHYH* | 602026 | Refsum disease | 0 |

SNV: Single Nucleotide Variation
